# Unraveling the Molecular Mechanisms of SIRT7 in Angiogenesis: Insights from Substrate Clues

**DOI:** 10.3390/ijms252111578

**Published:** 2024-10-28

**Authors:** Junjie Ma, Liqian Yang, Jiaxing Wu, Zhihong Huang, Jiaqi Zhang, Minghui Liu, Meiting Li, Jianyuan Luo, Haiying Wang

**Affiliations:** 1 Beijing Key Laboratory of Protein Posttranslational Modifications and Cell Function, Department of Biochemistry and Biophysics, School of Basic Medical Sciences, Peking University Health Science Center, Beijing 100191, China; 2110301354@stu.pku.edu.cn (J.M.); yangliqian1998@163.com (L.Y.); ida202207@163.com (J.W.); 2311110045@stu.pku.edu.cn (Z.H.); madeline_zhang7@163.com (J.Z.); luojianyuan@bjmu.edu.cn (J.L.); 2 Department of Medical Genetics, Center for Medical Genetics, Peking University Health Science Center, Beijing 100191, China; tsliumh@126.com (M.L.); limeiting@bjmu.edu.cn (M.L.)

**Keywords:** SIRT7, angiogenesis, substrate, molecular mechanism, vascular endothelial growth factor

## Abstract

Angiogenesis, a vital physiological or pathological process regulated by complex molecular networks, is widely implicated in organismal development and the pathogenesis of various diseases. SIRT7, a member of the Sirtuin family of nicotinamide adenine dinucleotide + (NAD^+^) dependent deacetylases, plays crucial roles in cellular processes such as transcriptional regulation, cell metabolism, cell proliferation, and genome stability maintenance. Characterized by its enzymatic activities, SIRT7 targets an array of substrates, several of which exert regulatory effects on angiogenesis. Experimental evidence from in vitro and in vivo studies consistently demonstrates the effects of SIRT7 in modulating angiogenesis, mediated through various molecular mechanisms. Consequently, understanding the regulatory role of SIRT7 in angiogenesis holds significant promise, offering novel avenues for therapeutic interventions targeting either SIRT7 or angiogenesis. This review delineates the putative molecular mechanisms by which SIRT7 regulates angiogenesis, taking its substrates as a clue, endeavoring to elucidate experimental observations by integrating knowledge of SIRT7 substrates and established angiogenenic mechanisms.

## 1. Introduction

Angiogenesis is the process of generating new capillaries from preexisting ones, with several morphological processes categorized into different angiogenic modes [1]. In embryos, angioblasts differentiate from the mesoderm and form primitive blood vessels, a process known as vasculogenesis [2]. In contrast, sprouting angiogenesis refers to vessel formation from a preexisting capillary bed [1]. Endothelial cells (ECs), which form a single layer lining the entire vascular system and whose structural and functional integrity are vital for vessel wall maintenance [3], are the key cells in angiogenesis [1]. Angiogenesis is a series of continuous and complex processes, mainly consisting of EC activation, extension, and quiescence recovery, regulated by numerous signaling pathways and molecules, including vascular endothelial growth factor (VEGF)/vascular endothelial growth factor receptor (VEGFR) signaling pathway, transforming growth factor-β (TGF-β) signaling pathway, NOTCH signaling pathway, angiopoietin (ANG)/TIE signaling pathway, fibroblast growth factor (FGF), placental growth factor (PlGF), platelet-derived growth factor (PDGF), matrix metalloproteinases (MMPs), neuropilins (NRP), chemokines, and so on [4].

Sirtuins (SIRT1-7), mammalian homologues of NAD^+^-dependent histone deacetylase, Sir2 in yeast, play pivotal roles in a wide array of biological and cellular processes, including metabolism, mitochondrial functions, oxidative stress response, DNA repair, apoptosis, senescence, and inflammation [5]. As a multifunctional enzyme with deacetylase, desuccinylase, defatty-acylase, debutyrylase, deglutarylase, decrotonylase, mono-adenosine 5′-diphosphate (ADP)-ribosyltransferase, and an NAD^+^-independent RNA deacetylase, SIRT7 is involved in various cellular processes such as transcriptional regulation, cellular metabolism, cell proliferation, and the maintenance of genome stability [6]. In recent years, accumulating evidence has underscored the regulatory role of SIRT7 in angiogenesis in different contexts [7,8,9,10,11,12,13]. However, the substrate-mediated molecular mechanisms remain unclear and fragmented. Thus, this review aims to provide a comprehensive overview of the molecular mechanisms underlying the regulation of angiogenesis by SIRT7, while considering substrates of SIRT7 as the clue (Table 1).

**Table 1:** Regulation of SIRT7 on angiogenesis mediated by substrates [14,15].

(Potential) Cell Type of SIRT7 Modification	Substrate Name	Regulation of SIRT7 on Its Substrates	Impact of SIRT7 on Its Substrates	Pathways or Molecules Involved in Regulating Angiogenesis	Angiogenic Effects of Substrates	Theoretical Effect of SIRT7 on Angiogenesis
*ECs*	*KLF4*	deacetylation [7]	increase stability [7]	*VEGF*/*VEGFR* signaling [16,17,18] Notch signaling [17,18]	promote [7,16,17,18]	promote
	*GABPβ1*	deacetylation [19]	increase activity [19]	*VEGF* [20] Robo4 [20]	inhibit [20]	inhibit
Non-endothelial normal tissue cells	GATA4	deacetylation [21,22]	reduce activity [21,22]	VEGFA [23,24] *ANG-4* [25]	promote [23,24,25]	inhibit
	*OSX*	deacetylation [26]	increase activity [26]	*VEGF* [27]	promote [27]	promote
	*PPARγ2*	deacetylation [28]	increase activity [28]	*VEGF*/*VEGFR* signaling and non-classical pathways ways reviewed in [29]	mainly inhibit [29]	mainly inhibit
	PICK1	not clear yet [11]	reduce activity [11]	*TGFBR1* [11]	inhibit [11]	promote
	p53	deacetylation [30]	reduce activity [30]	*HIF*-1α/*VEGF* axis [31]	inhibit [31]	promote
	*NFATc1*	deacetylation [32]	reduce stability [32]	*VEGF* [33,34]	promote [33,34]	inhibit
	CRY1	deacetylation [35]	reduce stability [35]	*VEGF* [36] *WEE1*, *HOXC5* [36]	promote [36]	inhibit
	Ran	deacetylation [37]	reduce activity [37]	*NF-κB* [37]	context-dependent	context-dependent
Tumor cells	p53	deacetylation [38]	reduce activity [38]	*VEGF* [39] inflammatory cytokines [40] *HIF*-1α/*VEGF* axis [41]	inhibit [39,40,41]	promote
	H3K18	deacetylation [13]	-	TIE2 [13]	inhibit after deacetylation [13]	inhibit
	*CHD1L*	deacetylation [12]	increase stability [12]	*HIF*-2α [12]	promote [12]	promote
	*PGK1*	deacetylation [42]	reduce stability [42]	not clear yet	inhibit [43]	promote
	*CDK9*	deacetylation [44]	increase activity [44]	*VEGF* [45] *STAT3*, *VEGF* [46]	promote [45,46]	promote
	FOXO3	deacetylation [47]	reduce activity [47]	VEGFA [48,49]	inhibit [48,49]	inhibit
	*PRMT5*	desuccinylation [50]	increase activity [50]	VEGFA [51] inflammatory cytokines [52] *HIF*-1α/*VEGFR*/Akt/*eNOS* axis [53,54]	promote [51,52,53,54]	promote
	*USP39*	deacetylation [55]	increase stability [55]	VEGFA165b [56]	promote [56]	promote
	SMAD4	deacetylation [57]	reduce stability [57]	not clear yet	not clear yet	not clear yet
	*FKBP51*	deacetylation [58]	increase activity [58]	*NF-κB*, IL-8 [59]	inhibit [59]	inhibit
	*ATM*	deacetylation [60]	reduce activity [60]	*HIF*-1/VEGFA [61]	promote [61]	inhibit

*EC* endothelial cell; *KLF4* Kruppel-like factor 4; *VEGF* vascular endothelial growth factor; *VEGFR* vascular endothelial growth factor receptor; *GABPβ1* GA-binding protein beta 1; *ANG-4* angiopoietin 4; *OSX* Osterix; *PPARγ2* peroxisome proliferator-activated receptor gamma 2; *TGFBR1* transforming growth factor receptor 1; *HIF* Hypoxia-inducible factor; *NFATc1* Nuclear factor of activated T cells c1; CRY1 Cryptochrome 1; *WEE1* WEE1 G2 checkpoint kinase; *HOXC5* homeobox C5; *NF-κB* Nuclear factor-κB; *CHD1L* chromodomain helicase/ATPase DNA binding protein 1-like gene; *PGK1* Phosphoglycerate Kinase 1; *CDK9* Cyclin-dependent kinase 9; *STAT3* signal transducer and activator of transcription 3; *PRMT5* protein arginine methyltransferase; *eNOS* endothelial nitric oxide synthase; *USP39* Ubiquitin-specific protease 39; *FKBP51* FK506 binding protein 51; IL-8 interleukin 8; *ATM* Ataxia-Telangiectasia Mutated.

## 2. SIRT7 Regulates Angiogenesis Through VEGF/VEGFR Signaling Pathway

As a central mediator of angiogenesis [62], the VEGF/VEGFR signaling pathway is the most well-studied classical signaling pathway with strict regulation at various levels [63]. Research evidence has shown that SIRT7 regulates the VEGF/VEGFR signaling pathway in ECs. In a cerebral ischemia-reperfusion injury model, both SIRT6 and SIRT7 upregulate VEGFA and VEGFR-2 in ECs, mediating the pro-angiogenic effects of trilobatin [9]. The therapeutic potential of the SIRT7/VEGFA/VEGFR2 axis in ECs is confirmed in another cerebral infarction model [10]. KLF4 (Kruppel-like factor 4) is a member of the zinc-finger transcription factor family [64], which shows consistent activation of the VEGF/VEGFR signaling pathway in various ECs [16,17,18]. Mechanistically, KLF4 upregulates VEGFA, VEGFR-1, and VEGFR-2 by binding to their promoters [17]. In turn, VEGFA enhances KLF4 succinylation and transcriptional activity mediated by GCN5, establishing a positive feedback loop [16]. In pulmonary artery endothelial cells (PAECs), SIRT7 deacetylates KLF4, preventing its ubiquitination and subsequent proteasomal degradation [7]. Thus, in ECs, SIRT7 potentially promotes the VEGF/VEGFR signaling pathway through KLF4 deacetylation. GA-binding protein (GABP) is a nuclear transcription factor consisting of two subunits, GABPα and either GABPβ or GABPγ [65]. In mouse neovascularized cornea, GABPα/β delivery suppresses VEGF expression in corneal ECs, thereby blocking angiogenesis [20]. SIRT7-mediated GABPβ1 deacetylation promotes its complex formation with GABPα, thereby enhancing transcriptional activation of the heterotetramer [19]. Consequently, SIRT7 may inhibit VEGF-induced angiogenesis by activating GABP.

SIRT7 also regulates VEGF expression in non-endothelial normal tissue cells. GATA4 belongs to the GATA family of zinc-finger transcription factors, playing a significant role in cardiocyte survival and apoptosis [66]. In mice following cryoinjury, cardiomyocyte-specific GATA4 knockout results in impaired myocardial angiogenesis, concomitant with decreased cardiocyte VEGFA expression [23]. At the molecular level, GATA4 directly binds to the VEGFA promoter and enhances its transcription [24]. SIRT7 deacetylates GATA4 and suppresses its transcriptional activity, thus counteracting cardiocyte hypertrophy [21]. Thus, it is conceivable that a myocardial-specific anti-angiogenic SIRT7/GATA4/VEGFA axis plays a significant role in cardiac angiogenesis. Osterix (OSX) is a zinc finger-containing transcription factor belonging to the SP/KLF family [67], which plays a significant role in VEGF-induced angiogenesis during bone formation. Mechanistically, in osteoblasts, OSX upregulates VEGF by binding to its native promoter [27]. SIRT7 deacetylates OSX, facilitating its depropionylation by SIRT1, and both modifications enhance its N-terminal transactivation activity [26]. Therefore, SIRT7 likely plays a role in regulating the VEGF/VEGFR signaling pathway through OSX, thereby regulating angiogenesis. The involvement of peroxisome proliferator-activated receptor gamma (PPARγ) in angiogenesis has been extensively summarized, particularly in pathological conditions. PPARγ predominantly inhibits angiogenesis by suppressing the expression of both VEGF and VEGFR [29]. However, in adipocytes, PPARγ promotes the expression of VEGFA and VEGFB, thereby promoting growth and tube formation of ECs, which leads to angiogenesis in adipose tissue [68]. SIRT7 deacetylates PPARγ2 and enhances its transcriptional activity on genes associated with adipogenesis in adipocytes [28]. It is plausible that in adipocytes, SIRT7 regulates VEGF/VEGFR-mediated angiogenesis through PPARγ2 activation. The nuclear factor of activated T cells c1 (NFATc1), a member of the NFAT family of transcription factors, operates downstream of the VEGF/VEGFR signaling [34,69]. In ECs, upon VEGF stimulation, NFATc1 dephosphorylates and translocates into the nucleus [34], where it transcriptionally regulates angiogenic genes [69], which is essential for in vitro VEGFA-induced angiogenic events, including proliferation, migration, sprouting, and tube formation [33]. SIRT7 deacetylates NFATc1 and promotes its proteasomal degradation in hair follicle stem cells [32], which suggests that SIRT7 may impede downstream signals of the VEGF/VEGFR signaling pathway. Cryptochrome 1 (CRY1) is a key regulator of circadian rhythms [70]. In a mouse hind limb ischemia model, circadian rhythm disruption impedes the expression of clock genes, including CRY1, leading to decreased capillary density and impaired tissue blood perfusion recovery, which are associated with VEGF downregulation [36]. SIRT7 deacetylates CRY1 and facilitates its degradation [35], by which SIRT7 potentially contributes to the inhibition of VEGF-induced angiogenesis.

In tumors, SIRT7 exhibits pro- or anti-tumor functions in a context-dependent manner [71]. Tumor suppressor p53 [72] impedes angiogenesis in tumor contexts by downregulating VEGF [73]. Specifically, in advanced hepatocellular carcinoma (HCC), p53 inhibits oxidative stress and angiogenesis, leading to reduced tissue VEGF levels and exerting anti-cancer effects [39]. SIRT7 deacetylates and inhibits p53 activity in HCC [74], maybe thereby modulating VEGF-induced angiogenesis. Cyclin-dependent kinase 9 (CDK9) is a prominent member of the transcriptional CDK subfamily [75], whose necessity in VEGF expression has been repeatedly confirmed by in vitro studies [45,46]. In non-small-cell lung cancer cells, inhibition of the CDK9/signal transducer and activator of transcription 3 (STAT3) signaling pathway suppresses the expression of STAT3 target genes, including VEGF, thereby inhibiting tumor growth [46]. SIRT7 deacetylates CDK9, which enhances phosphorylation of RNA polymerase II and activates transcription [44]. Thus, SIRT7 may promote VEGF-induced angiogenesis by CDK9 deacetylation. FOXO3 belongs to the conserved O subgroup of the forkhead box (FOX) transcription factor family [76]. In breast cancer cells, FOXO3a displaces FOXM1 and recruits histone deacetylase 2 (HDAC2) to the VEGF promoter, leading to reduced histone acetylation and suppressed VEGF transcription [48]. In addition, FOXO3a induces miR-29b-2 and miR-338 expression, which directly inhibits the transcription of VEGFA and NRP1 (a coreceptor of VEGFA), respectively, thereby inhibiting breast cancer metastasis [49]. In the Hela cells, SIRT7-mediated deacetylation results in decreased FOXO3 phosphorylation and impaired cellular apoptotic response to lipopolysaccharide (LPS) [47]. Furthermore, SIRT7 promotes HCC metastasis by inhibiting the transcriptional activity of FOXO3 [77]. Consequently, SIRT7 likely inhibits VEGF-induced angiogenesis through FOXO3 deactivation. PRMT5, the type II class of protein arginine methyltransferases (PRMTs) [52], plays a significant role in promoting VEGF/VEGFR signaling and subsequent angiogenesis. In human glioma, PRMT5 facilitates the enrichment of H3R2me1 and H3R2me2s, recruiting WD repeat domain 5 (WDR5) to promote H3K4 methylation at the VEGFA promoter, thereby enhancing its transcription [51]. SIRT7 catalyzes the desuccinylation of PRMT5, thereby augmenting its methyltransferase activity and inhibiting its ubiquitination [50], thereby promoting lipid metabolic reprogramming, tumor growth, and tumor metastasis in HCC. Thus, SIRT7 may promote VEGF/VEGFR-induced angiogenesis. Ubiquitin-specific protease 39 (USP39), a deubiquitination enzyme [78], enhances vascular EC tube formation in clear cell renal cell carcinoma (RCC) by downregulating VEGFA165b, an endogenous splice isoform that inhibits VEGFA165 [79], though without significantly affecting overall VEGFA expression [56], which promotes the proliferation of the RCC. In HCC, SIRT7-mediated deacetylation impedes MYST1-facilitated acetylation of USP39, which is a prerequisite for its E3 ligase-induced degradation [55] and accelerates the tumorigenesis of HCC. Consequently, SIRT7 may hinder VEGFA-induced angiogenesis by preventing USP39 degradation. In summary, SIRT7 extensively modifies substrates in tumor cells, which may regulate the VEGF/VEGFR signaling pathway in tumor cells, modulating tumor angiogenesis.

## 3. SIRT7 Regulates Angiogenesis Through TGF-β Signaling Pathway

The TGF-β signaling pathway functions extensively across diverse cell types in numerous cellular responses encompassing survival, metabolism, growth, proliferation, differentiation, adhesion, migration, and apoptosis [80]. SMAD proteins serve as intermediaries in transducing extracellular signals to the nucleus [81], which form receptor-regulated SMADs (R-SMADs) to regulate target genes in collaboration with transcription factors and chromatin remodeling proteins [82]. While previous studies have summarized the dual effects of the TGF-β signaling pathway in angiogenesis [4], recent reviews have focused specifically on its pro-angiogenic role [83], particularly in tumor contexts.

In mouse models of myocardial infarction, hindlimb ischemia and skin injury, SIRT7 deficiency impedes angiogenesis in the corresponding regions. In the cardiac fibroblasts, SIRT7 controls TGFBR1 degradation by interacting with PICK1 [11]. Moreover, SIRT7 also has the potential to regulate angiogenesis through TGF-β signaling pathway in breast cancer. In breast cancer cells, SIRT7 deacetylates SMAD4 and facilitates its degradation, which inhibits TGF-β signaling and subsequent breast cancer invasion and migration [57], which is possibily associated with tumor angiogenesis. Collectively, these findings underscore the significance and potential of SIRT7 as a critical regulator in the TGF-β signaling pathway, with profound implications in angiogenesis.

## 4. SIRT7 Regulates Angiogenesis Through Notch Signaling Pathway

The Notch signaling cascade is a conserved pathway that intricately governs cellular fate via intercellular communication [84]. Notch, encompassing Notch1–4 in mammals, serve as transmembrane receptors, engaging with ligands including Jagged 1 (JAG1), JAG2, delta-like ligand 1 (Dll1), Dll3, and Dll4, which ultimately orchestrate the transcription of downstream target genes [85]. Through nuanced regulation of various EC activities, Notch signaling assumes a central role in angiogenesis [85,86].

Current evidence does not substantiate direct regulation of Notch signaling by SIRT7, but indirect regulation exists. As previously noted, KLF4 is deacetylated and stabilized by SIRT7 in PAECs [7]. In an endothelium-specific KLF4 overexpression model, KLF4 differentially regulates the expression of Notch receptors, ligands, and target genes, and constrains cleavage-mediated Notch1 activation, thereby promoting invalid angiogenesis with heightened vessel density but diminished perfusion [17]. Further mechanistic investigations indicate that in ECs, KLF4 inhibits Dll4 expression by disrupting the formation of the recombination signal binding protein for immunoglobulin kappa J region (RBPj)-NICD-mastermind-like (MAML) complex in Dll4 intron [18]. In summary, through its modification on KLF4, SIRT7 serves as a key participant in Notch signaling-mediated angiogenesis.

## 5. SIRT7 Regulates Angiogenesis Through ANG/TIE Signaling Pathway

The ANG/TIE signaling pathway is identified as the second EC-specific receptor tyrosine kinase signaling system, following the discovery of the VEGF/VEGFR system [87]. TIE1 and TIE2 are two ANG receptors predominantly expressed in the endothelium, while ANG1–4 serve as ligands [88]. The ANG/TIE signaling pathway plays a pivotal role in regulating blood vessel stability, angiogenesis, and inflammation [89]. Detailed insights into this pathway have been extensively reviewed [87,88,89].

In breast cancer cells, SIRT7 deacetylates H3K18 at the TIE2 promoter and suppresses its transcription, thereby inhibiting adriamycin-induced angiogenesis in breast cancer, as well as invasion and metastasis [13]. Additionally, in cardiocytes, SIRT7 deacetylates and deactivates cardiogenic transcription factors GATA4 [21], as mentioned above, while GATA4/6 deletion in cardiac fibroblasts promotes the expression of ANG-4 and other anti-angiogenic genes to impedes cardiac angiogenesis [25]. Thus, in the heart, SIRT7 has a huge potential to regulate angiogenesis by ANG/TIE signaling pathway. Overall, SIRT7 is an important element in ANG/TIE-mediated angiogenic events, exerting its regulatory role through deacetylation.

## 6. SIRT7 Regulates Angiogenesis Through Inflammation

Inflammation is a multifaceted process comprising a complex network of interconnected mechanistic pathways, initially serving as a protective response but leading to disorders when dysregulated [90]. Due to its extreme complexity and diversity, there is currently no universal yet comprehensive model of inflammation. However, context-specific inflammatory responses have been well depicted [91]. Angiogenesis is intricately intertwined with inflammation in various pathological conditions [92].

ECs are not only the protagonists of angiogenesis [1] but also indicators and mediators in inflammation, whose dysregulation or dysfunction leads to vascular dysfunction and structural abnormalities, marking the initial step in the pathogenesis of vascular inflammatory disorders [90]. In the Hutchinson–Gilford progeria model, ectopic expression of SIRT7 in regenerated vascular ECs results in increased angiogenesis. At the molecular level, SIRT7 demonstrates inhibitory effects on EC-produced inflammatory cytokines, including chemokine C-C motif ligand 2 (CCL2), CCL4, IL-1β, IL-6, SERPINE2, TGF-β2, TNF-α, and vascular cell adhesion molecule 1 (VCAM-1) [8]. Intriguingly, these cytokines predominantly promote angiogenesis in ECs (Table 2). However, as shown in Table 2, the angiogenic effects of these cytokines are almost singly validated, which may not correspond to the overall inhibitory function of SIRT7.

**Figure 1:**
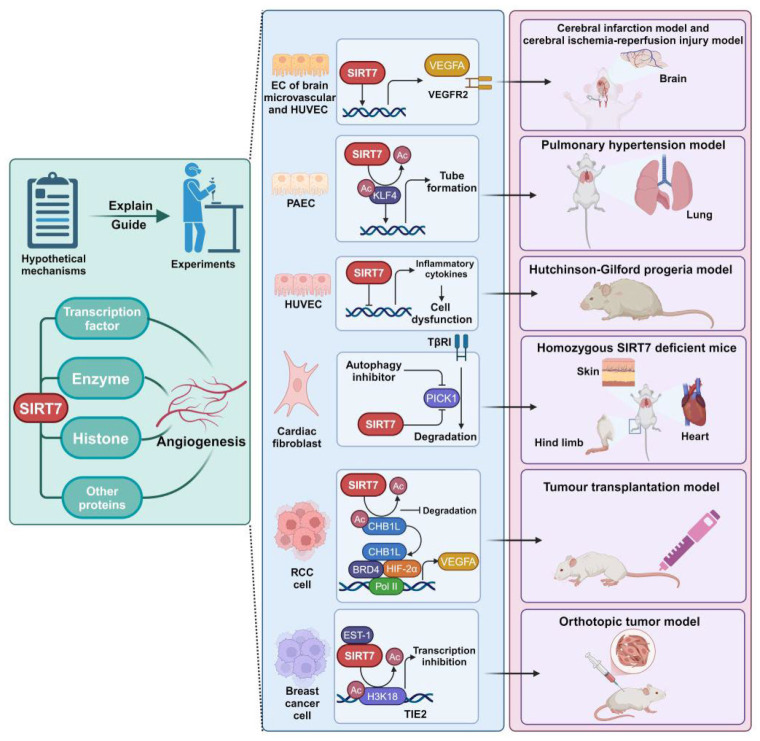
Summary of this review. Further exploration into the hypothetical mechanisms underlying the angiogenic regulation of SIRT7 not only elucidates previous experimental findings but also informs the design of future experiments. SIRT7 orchestrates modifications on its substrates, thereby regulating angiogenesis via multiple angiogenic signaling pathways. Various studies have demonstrated the role of SIRT7 in modulating angiogenesis in both in vitro and in vivo models. (Created with BioRender.com).

Nuclear factor-κB (NF-κB), a highly conserved transcription factor family, which includes REL (c-REL), RELA (p65), RELB, NFκB1 (p50), and NFκB2 (p52), regulates the transcription of diverse genes and participates in various activities, including both inflammation and angiogenesis. During pathological angiogenesis, NF-κB signaling demonstrates context-dependent regulation by either activating ECs and stimulating basement membrane degradation or deactivating ECs and preventing extracellular matrix degradation [93]. Four substrates potentially mediate SIRT7 regulation of NF-κB signaling, namely p53, PRMT5, FKBP51, and Ran (Figure 1). P53, deacetylated and deactivated by SIRT7 in HCC [74], possesses widely recognized inflammatory roles and abundant context-dependent regulation on NF-κB [40]. In HCC, the p53 pathway and the NF-κB pathway crosstalk to promote autophagy and inhibit apoptosis [94]. The pro-angiogenic role of NF-κB in HCC has been repeatedly confirmed, and it is considered both an anti-cancer target and a negative prognostic indicator [95,96]. In HCC, SIRT7 desuccinylates, thereby activating and stabilizing PRMT5 [50], where the latter is essential for NF-κB activity and the expression of its target genes, including tumor necrosis factor-alpha (TNF-α) and VEGFA in neovascular age-related macular degeneration and laser-induced choroidal neovascularization models [52]. In breast cancer cells, FK506 binding protein 51 (FKBP51), a member of the FKBPs [97], is deacetylated and activated by SIRT7 [58]. It enhances NF-κB activity and subsequently upregulates IL-8 to promote melanoma growth, metastasis, and angiogenesis in melanoma [59]. Ran, a member of the Ras superfamily, undergoes dynamic cycling between Ran GTP and Ran GDP states and serves as a regulator of normal cellular function [98]. SIRT7 deacetylates Ran, which impedes the interaction between Ran as well as p65 and chromosome region maintenance protein 1 (CRM1), thereby blocking the nuclear export of p65 [37]. Altogether, SIRT7 plays a crucial role in regulating inflammation, which may further influence angiogenesis.

**Table 2:** SIRT7 regulates angiogenesis through inflammatory cytokines in ECs [8].

Inflammatory Cytokines	Pro-Angiogenic Signaling Pathways	Anti-Angiogenic Signaling Pathways	Regulation on Angiogenesis
*CCL2*	*STAT3* [99] *STAT3*, AKT [100] *MT1-MMP* [101]	-	promote
*TNF-α*	*VEGF*/AKT/p38 [102]	-	promote
*VCAM-1*	*JunB*, *IL*-8 [103] *EGR-3*/*CREB*/*VEGF* [104]	-	promote
*IL*-1β	*TEM-8* [105] *VEGF*, *CXCL2*, etc. [106]	fibrillar actin [107]	context-dependent
*IL*-6	survivin/caspase-3 and -7 [108] *DNMT1*, VEGFR-2 [109] *CaMKIIδ* [110]	*CXCL10*, SERPINF1, *Ckit*, *CXCL8*, etc. VEGFA [111]	context-dependent
*CCL4*	not clear yet		
SERPINE2	not clear yet		
*TGF-β2*	not clear yet		

*CCL2* chemokine C-C motif ligand 2; *STAT3* signal transducer and activator of transcription 3; *MT1-MMP* membrane type 1 matrix metalloproteinase; *TNF-*α tumor necrosis factor; *VEGF* vascular endothelial growth factor; *VCAM-1* vascular cell adhesion molecule 1; *JunB* JunB proto-oncogene; *IL* interleukin; *EGR-3* early growth responsive gene 3; *CREB* cyclic AMP response element-binding protein; *TEM-8* tumor endothelial marker 8; *CXCL2* C-X-C motif chemokine ligand 2; *DNMT1* DNA methyltransferase 1; *VEGFR-2* vascular endothelia growth factor receptor 2; *CaMKIIδ* Ca^2+^/calmodulin-dependent protein kinase II; *CXCL* C-X-C motif chemokine ligand; *Ckit* KIT proto-oncogene; *CCL4* chemokine C-C motif ligand 4; *TGF*-β2 transforming growth factor β2.

## 7. SIRT7 Regulates Angiogenesis Through HIF

The hypoxia-inducible factor (HIF) transcription factor, consisting of constitutive HIF-1β and either HIF-1α or HIF-2α isoforms, is the primary regulator of the hypoxia response [112]. HIF-1 especially upregulates VEGF in ischemic-hypoxic tissues and ECs [113]. SIRT7 indirectly regulates HIF-mediated angiogenesis through its substrates. For example, the chromodomain helicase/ATPase DNA binding protein 1-like gene (CHD1L), belonging to the sucrose non-fermentation 2 (SNF2) family, is a chromosome regulator, transcription and translation activator [114]. In RCC, CHD1L interacts with HIF-2α to enhance its transcriptional activity, recruits bromodomain containing 4 (BRD4), and augments phosphorylation of RNA polymerase II, which amplifies hypoxia signals, such as VEGFA, leading to increased cellular proliferation, migration, and self-renewal capacity. SIRT7 stabilizes CHD1L by facilitating its deacetylation and attenuating its ubiquitination [12]. As mentioned above, p53, deacetylated by SIRT7 in HCC [74], impedes the binding of HIF-1α and p300, thereby downregulating VEGF expression and inhibiting angiogenesis in RCC [41]. In addition, in the diabetic cardiomyopathy model, inhibition of p53 prevents cardiac angiogenic defects by stabilizing HIF-1α and upregulating the transcription of specific target genes, including VEGF and VEGFR2 [31]. In cardiocytes, SIRT7 deacetylates p53, increasing cellular stress resistance [30]. Ataxia-Telangiectasia Mutated (ATM), a member of the phosphoinositide 3-kinase (PI3K)-related protein kinase (PIKK) family [115], mediates phosphorylation of seryl-tRNA synthetase (SerRS), which alleviates its transcriptional inhibition on hypoxia-induced binding of c-Myc and HIF-1 to the VEGFA promoter, and expression activation of VEGFA, thereby inducing angiogenesis in breast cancer [61]. The ATM/HIF1α signaling is also demonstrated in lung cancer [116]. Noticeably, in human colon cancer cells, SIRT7-mediated deacetylation of ATM is crucial for its dephosphorylation and subsequent deactivation [60]. Moreover, PRMT5, desuccinylated by SIRT7 [50], maintains the activation and stabilization of HIF-1α, which is essential to the phosphorylation of VEGFR2 as well as downstream Akt and endothelial nitric oxide synthase (eNOS) in lung cancer cells and ECs, playing a significant role in angiogenesis [53,54]. In all, SIRT7 emerges as a pivotal regulator in HIF-induced angiogenesis.

## 8. SIRT7 Regulates Non-Classical or Unclear Angiogenic Signaling Pathways

Angiogenesis is a complex process involving a myriad of molecules and signaling cascades [4]. Beyond the seven established angiogenic pathways delineated earlier, both in vivo and in vitro investigations have underscored the role of SIRT7 in angiogenesis through a diverse spectrum of non-canonical angiogenic signaling pathways. Furthermore, certain instances of substrate-mediated angiogenic regulation have been documented, but without a clear elucidation of the downstream molecular mechanisms (Table 3).

## 9. Conclusions and Perspectives

Theoretically, through posttranslational modifications of substrates mediated by SIRT7, a complex angiogenic network is established, in which convincing experimental evidence has demonstrated the effects of SIRT7 in modulating angiogenesis in various contexts [7,8,9,10,11,12,13] (Figure 1). This review, structured around substrates, elucidates numerous potential molecular mechanisms through which SIRT7 regulates angiogenesis. Furthermore, the practical significance lies in validating these mechanisms in specific cellular contexts and physiological or pathological states, both in vitro and in vivo. In addition, though it is rational to prioritize EC as the primary research model for dissecting these angiogenic mechanisms, it is imperative to emphasize the elaborate cell-to-cell and cell-microenvironmental interplay as well as long-range cellular communication. Furthermore, drawing parallels from studies on other members of the Sirtuin family could provide valuable insights due to their similarities [117].

## Figures and Tables

**Table 3:** SIRT7 substrates regulate angiogenesis through non-classical angiogenic signaling pathways.

Substrate Name	Downstream Molecules	Angiogenic Effects of Substrates	In Vivo Model	In Vitro Model	Reference
*KLF4*	not clear yet	promote	endothelium-specific SIRT7-KO mouse, *PH* mouse model	*PAECs*	[7]
*GABPβ1*	Robo4	promote	mouse model of corneal neovascularization	human conjunctival *ECs*	[20]
*CRY1*	*WEE1*	promote	hindlimb ischemia model	HUVECs	[36]
	*HOXC5*	promote			
PPARγ2	*ANGPTL4*	promote	mouse model	adipocytes	[68]
*PGK1*	not clear yet	inhibit	mouse subcutaneously tumor model, mouse liver metastasis tumor model	*HCC* cells	[43]

*KLF4* Kruppel-like factor 4; *PH* pulmonary hypertension; *PAEC* pulmonary artery endothelial cell; *GABPβ1* GA-binding protein beta 1; *EC* endothelial cell; CRY1 Cryptochrome 1; *WEE1* WEE1 G2 checkpoint kinase; *HOXC5* homeobox C5; *HUVEC* human umbilical vein endothelial cell; *PPARγ2* peroxisome proliferator-activated receptor gamma 2; *ANGPTL4* Angiopoietin-like 4; *PGK1* Phosphoglycerate Kinase 1; *HCC* hepatocellular carcinoma.
